# Perception and success rate of using advanced airway management by hospital-based paramedics in the Kingdom of Saudi Arabia

**DOI:** 10.29045/14784726.2021.12.6.3.24

**Published:** 2021-12-01

**Authors:** Amani Alenazi, Bashayr Alotaibi, Najla Saleh, Abdullah Alshibani, Meshal Alharbi, Nawfal Aljerian, Nesrin Alharthy, Sameerah Alsomali

**Affiliations:** King Saud bin Abdulaziz University for Health Sciences (KSAU-HS); Ministry of Health, Saudi Arabia; Ministry of Health, Saudi Arabia; King Saud bin Abdulaziz University for Health Sciences (KSAU-HS); King Saud bin Abdulaziz University for Health Sciences (KSAU-HS); Ministry of Health, Saudi Arabia; King Saud bin Abdulaziz University for Health Sciences (KSAU-HS); King Saud bin Abdulaziz University for Health Sciences (KSAU-HS)

**Keywords:** ambulance service, mechanical ventilation, pre-hospital care, supraglottic airway devices, tracheal intubation

## Abstract

**Objective:**

The study aimed to measure the success rate of pre-hospital tracheal intubation (TI) and supraglottic airway devices (SADs) performed by paramedics for adult patients and to assess the perception of paramedics of advanced airway management.

**Method:**

The study consisted of two phases: phase 1 was a retrospective analysis to assess the TI and SADs’ success rates when applied by paramedics for adult patients aged >14 years from 2012 to 2017, and phase 2 was a distributed questionnaire to assess paramedics’ perception of advanced airway management.

**Result:**

In phase 1, 24 patients met our inclusion criteria. Sixteen (67%) patients had TI, of whom five had failed TI but then were successfully managed using SADs. The TI success rate was 69% from the first two attempts compared to SADs (100% from first attempt). In phase 2, 63/90 (70%) paramedics responded to the questionnaire, of whom 60 (95%) completed it. Forty-eight (80%) paramedics classified themselves to be moderately or very competent with advanced airway management. However, most of them (80%) performed only 1–5 TIs or SADs a year.

**Conclusion:**

Hospital-based paramedics (i.e. paramedics who are working at hospitals and not in the ambulance service, and who mostly respond to small restricted areas in Saudi Arabia) handled few patients requiring advanced airway management and had a higher competency level with SADs than with TI. The study findings could be impacted by the low sample size. Future research is needed on the success rate and impact on outcomes of using pre-hospital advanced airway management, and on the challenges of mechanical ventilation use during interfacility transfer.

## Introduction

Emergency medical services (EMS) is a system that provides pre-hospital care to all emergency cases (Alanazi, 2012). EMS personnel are trained to handle all medical and trauma emergency cases through ensuring personal safety, performing rapid and accurate on-scene assessment, providing high-quality initial management and appropriately triaging and transporting patients (Al Mutairi et al., 2016; Alanazi, 2012). There are two EMS levels in the Kingdom of Saudi Arabia (KSA): 1) Basic life support (BLS), which is mainly focused on providing basic care such as oxygen administration and cardiopulmonary resuscitation, and 2) Advanced life support (ALS), which provides both basic and advanced care such as tracheal intubation (TI) and drug administration (Al Mutairi et al., 2016; Alanazi, 2012).

The Saudi Red Crescent Authority (SRCA) is the established governmental agency to provide pre-hospital emergency care in KSA (Al Mutairi et al., 2016). It is responsible for responding to all emergency cases across KSA, including cases in rural and urban regions, with its ability to provide BLS and ALS care from qualified personnel (Al Mutairi et al., 2016; AlShammari et al., 2017). In addition to SRCA, there are other healthcare facilities in the kingdom that have EMS stations and provide pre-hospital care such as King Abdulaziz Medical City (KAMC) (military hospital), King Fahad Medical City (KFMC) (general Ministry of Health [MOH] hospital) and the King Faisal Specialist Hospital and Research Center (KFSHRC) (specialist MOH hospital) (AlShammari et al., 2017; Khattab et al., 2019).

To clarify the structure of the EMS system in KSA: the role of the SRCA is to respond initially to all emergency cases across the kingdom while the role of hospital-based EMS in initial response is restricted to small regions. For example, the EMS of military hospitals respond to emergency cases in military residential compounds. For Interfacility Transfers (IFT), it is totally dependent on hospital-based EMS, whereas the SRCA has no involvement in such transfers (AlShammari et al., 2017; Khattab et al., 2019).

Pre-hospital airway management is one of the essential emergency skills and competencies for EMS personnel worldwide, as it was shown to be a lifesaving procedure in critical emergency cases (Jacobs & Grabinsky, 2014; Rognås et al., 2013). Different modalities are currently used in pre-hospital care, including supraglottic airway devices (SADs) and endotracheal tube (ETT) (Gupta et al., 2015; Wang et al., 2011). One of the quality measures for successful pre-hospital airway management is the rate of successful TI (Wang et al., 2011). However, there is no clear evidence about the benefits of pre-hospital TI, and some other studies have argued that it may cause more harm than good (Pepe et al., 2015). Its successful application in pre-hospital care needs high-quality training, education and clear protocol to improve outcomes (Tiah et al., 2014). Limited TI experience for EMS personnel was shown to be associated with increased odds of mortality (Odds Ratio 2.33, 95% Confidence Interval 1.61–3.38, p < 0.001) (Bossers et al., 2015). Alternatively, SADs are less invasive and require minimal training compared with TI such as laryngeal mask airway (LMA), LT King and Combitube (Jacobs & Grabinsky, 2014). They can be used as a backup or rescue device for failed face mask ventilation and/or TI (Gordon et al., 2017). However, using SADs is associated with complications in some cases, such as aspiration and airway trauma (Gordon et al., 2017; Jacobs & Grabinsky, 2014).

To our knowledge, no study has yet measured the perception and success rate of pre-hospital advanced airway management in KSA. This study, therefore, aimed to measure the success rate of pre-hospital TI and SADs and to assess paramedics’ perception about pre-hospital airway management.

## Methods

### Study design

This study mainly consisted of two phases: a retrospective analysis, and a distributed questionnaire for paramedics at KAMC, KFMC and KFSHRC in Riyadh, KSA. Ethical approval from King Abdullah International Medical Research Center (KAIMRC) was obtained for both phases. In phase 1, data collection and analysis were performed for all adult patients aged >14 years who received advanced airway management by paramedics, to measure the success rate of pre-hospital TI and SADs. Based on the information we collected from the KAMC’s database, all patients above 14 years are considered adults.

Data were collected from 1 January 2012 to 30 September 2017. In phase 2, a questionnaire was distributed to all paramedics working at KAMC, KFMC and KFSHRC to assess their perception of pre-hospital advanced airway management. A participant information sheet was provided, and signed consent forms were collected from all participants where they agreed to share their anonymised information.

### Study subjects

In phase 1, adult patients aged >14 years who required pre-hospital advanced airway management and were transported through EMS at KAMC, KFMC and KFSHRC between January 2012 and September 2017 were included in the study. Exclusion criteria include patients transported through SRCA, patients dead on arrival and incomplete reports. Phase 2 included all paramedics working at KAMC, KFMC and KFSHRC who responded and completed the questionnaires and excluded those who did not.

### Data collection

In phase 1, patients’ medical records were obtained from the EMS department registry. The patients were grouped by year from January 2012 to September 2017. Demographics of the patients were collected from the charts, including age, sex and indication for TI. The primary outcome measure was the success rate of TI and SADs performed by paramedics. Some other important variables were also collected, including the numbers of attempts for TI and SADs and the type of applied SADs.

In phase 2, data were collected using a questionnaire which was distributed to paramedics (Supplementary 1). The questionnaire was written in English language only and was validated using a face-validity method as it was distributed to a panel of paramedics and emergency physicians to get their feedback. The questionnaire was also checked by the panel for its reproducibility.

### Data management and analysis

All collected anonymised raw data from medical records including demographic information and other variables were compiled using Microsoft Excel (2016). They were then exported to IBM SPSS Statistical (IBM Corp., Armonk, NY, USA) software version 22 for analysis. Descriptive statistics were reported in frequencies and percentages for categorical data. The Pearson Chi-Square test was used for the comparison.

For missing data, we set a priori that if the missing data of the total observations in phase 1 are less than 5%, they will be deleted as the rule of thumb indicated that missing data with this percentage can be deleted without any significant ramifications (Harrell, 2001). If they were >5%, multiple imputations were predetermined to be appropriate. In phase 2, incomplete questionnaires were predetermined to be excluded from the analysis. After obtaining the data, no missing data were identified in phase 1, and three (5%) questionnaires in phase 2 were excluded as they were incomplete. In order to minimise bias, we predetermined clear inclusion/exclusion criteria and how to handle missing data for both phases.

## Results

### Demographic data

In phase 1, 24 patients were included from only one hospital (KAMC), of whom 54% were female. The median age was 66.5 years and ranged from 16 to 108 years. Although KFMC and KFSHRC have qualified paramedics and clear protocols to perform advance airway management, no case requiring pre-hospital advanced airway management was encountered during the data collection period in either centre.

In phase 2, the questionnaire was distributed to 90 paramedics over all three hospitals. Sixty-three (70%) paramedics responded to the questionnaire, of whom 60 (95%) completed it. The results showed that 53% of the paramedics were aged between 20 and 30 years, and most of them were male (95%). Forty-nine (82%) paramedics held a Bachelor’s degree. Two of them held a diploma degree, but they were registered as paramedics by the Saudi Commission for Health Specialties. Twenty-eight (47%) paramedics had <5 years of experience.

### Success rate of pre-hospital advanced airway interventions

**Figure 1:**
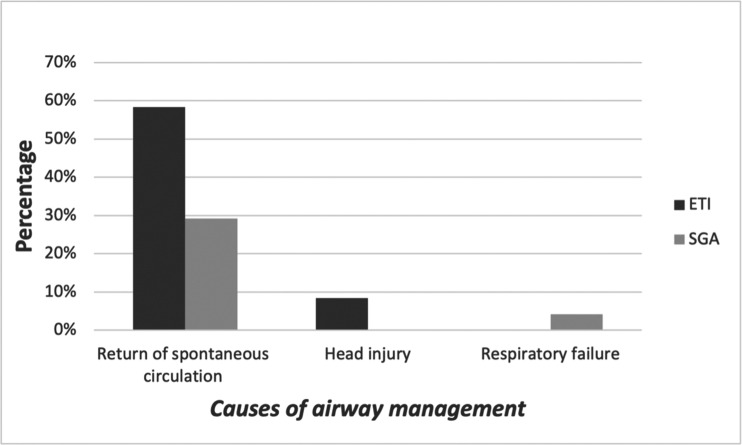
Figure 1. The percentages of using TI versus SADs in patients with different medical conditions.

Twenty-one (88%) cases required pre-hospital advanced airway management, mainly due to cardiac arrest (Figure 1). TI was the first choice for advanced airway management in 16/24 (67%) cases, compared to SADs in 8/24 cases (33%). Using TI was successful in 11/16 (69%) cases from the first two attempts, and 5/16 (31%) cases were failed TI but then alternatively and successfully managed using SADs. In the SADs group, all patients were successfully managed from the first attempt (100%). Among the SADs group, the LT King device was used in 8/13 (62%) patients and LMA in 5/13 (38%) patients.

The Pearson Chi-Square test was used to assess the relation between success rate and number of intubation attempts, as well as between success rate and age. There was no significant difference between different age groups and success rate or between number of intubation attempts and success rate (p = 0.68 and 0.48, respectively). The nature of emergency cases requiring advanced airway management did not affect the paramedics’ judgement in applying different advanced airway management devices (p = 0.52).

### Perception about pre-hospital advanced airway management

Fifty-three (88%) paramedics argued that their study programmes provided them with enough comprehensive knowledge and skills to manage difficult airways. Thirty-four (57%) paramedics had continuous educational development in airway management at their place of work. Thirty (50%) took different advanced airway courses (Table 1). Moreover, 57 (95%) paramedics agreed that airway management skills should be formally assessed.

**Table 1.** Advanced airway courses taken by paramedics in all hospitals.

Advanced airway courses	Number of paramedics				Continuing Medical Education (CME) hours	Institution
	KAMC	KFMC	KFSHRC	Total		
**From intubation to mechanical ventilation**	4	3	-	7	14	KFMC
**Advanced airway management**	6	1	2	9	–	Prince Sultan Military Medical City
**Critical care course**	2	–	–	2	–	KAMC
**Airway intervention and management in emergency**	2	–	–	2	–	–
**Tracheostomy management**	–	–	3	3	–	–
**Airway management**	2	1	4	7	5	KAMC
					–	KFMC
**Total**	16	5	9	30		

Of all the participants in this questionnaire, 48/60 (80%) classified themselves to be moderately or very competent with advanced airway management. Seventy-eight percent encountered difficult airway cases, and the majority (80%) performed 1–5 TIs or SADs per year. Most (63%) of the paramedics preferred the use of LMA to other SADs because they are easier, more comfortable, save time and are associated with fewer complications (Table 2). Level of paramedic education and years of experience made no significant difference to competency level, with a p-value of 0.97 and 0.60 respectively based on the Pearson Chi-Square test.

**Table 2.** Paramedics’ demographic data.

Variables	KAMC	KFMC	KFSHRC	Total
	N = 23(38%)	N = 20(33%)	N = 17(28%)	N = 60(100%)
**Level of education:**				
Diploma	2(9%)	0(0%)	0(0%)	2(3%)
Bachelor’s	17(74%)	17(85%)	15(88%)	49(82%)
Master’s	4(17%)	3(15%)	2(12%)	9(15%)
**Years of experience:**				
<5 years	7(30%)	13(65%)	8(47%)	28(47%)
5–10 years	6(26%)	2(10%)	4(24%)	12(20%)
>10 years	10(44%)	5(25%)	5(29%)	20(33%)
**Level of competency in dealing with patients who require advance airway management:**				
Not competent	1(4%)	0(0%)	2(12%)	3(5%)
Slightly competent	4(17%)	3(15%)	2(12%)	9(15%)
Moderately competent	11(48%)	7(35%)	6(35%)	24(40%)
Very competent	7(30%)	10(50%)	7(41%)	24(40%)
**Preferred supraglottic airway device:**				
LT King	16(70%)	0(0%)	3(18%)	19(32%)
LMA	6(26%)	19(95%)	13(76%)	38(63%)
Combitube	1(4%)	1(5%)	0(0%)	2(3%)
I-gel	0(0%)	0(0%)	1(6%)	1(2%)

## Discussion

The advanced airway management by the paramedics in our study is considered to be unique in terms of success rate compared to international findings, as well as in terms of the high success rate using SADs. In phase 1, our study showed that TI was initially used more frequently by paramedics than SADs. Using TI had a 69% success rate from the first two attempts, and failed TI cases were alternatively and successfully managed using SADs. Applying SADs was 100% successful from the first attempt. In phase 2, this study found that most hospital-based paramedics encountered 1–5 difficult airway cases each year. It also showed that 50% of them completed advanced airway management courses. Despite their different levels of experience, most of these paramedics ranked themselves to be moderately or very competent in managing patients who required advanced airway management.

Previous literature assessed pre-hospital success rates of TI and SADs from the first attempt and found no significant difference (67% vs 68%, respectively) (Frascone et al., 2011). This contrasts with our findings which showed more difference in success rates (69% for TI and 100% for SADs). However, our study had a small sample size (n = 24) compared to this study (n = 204). Findings from two large trials in the UK and USA found significant differences in the success rate of SADs vs. TI (Benger et al., 2018; Wang et al., 2011). Benger et al. (2018) showed a higher success rate with initial ventilation using SADs compared to using TI (87.4% vs. 79.0%, respectively). Wang and colleagues (2011) found a 90.3% initial success rate when using LT compared to 51.6% when using TI (excluding bag-valve mask ventilation).

In our study, most paramedics had low intubation experience (1–5 cases each year). This might be due to the role of hospital-based EMS in initial response in KSA, which is restricted to small regions, hence fewer patients require pre-hospital care, including those who require advanced airway management. An intubation experience of at least 50 intubations was estimated for achieving a proficiency of ≥90% success rate (Buis et al., 2016). However, it would take years for our paramedics to achieve this level of experience. Our findings showed that most cases that required pre-hospital advanced airway management were cardiac arrest cases. Previous intubation experience for paramedics in such cases was associated with successful TI, which can be confirmed by using waveform capnography or end-tidal CO2 detectors (Dyson et al., 2017). It was suggested, therefore, that the application of SADs might be more appropriate for paramedics with little intubation experience (Dyson et al., 2017). However, it is still uncertain if using SADs and TI achieves equivalent outcomes. In our study, most SADs were applied for cardiac arrest cases, except for one case which had difficulty breathing. The reasons for paramedics’ decisions to use TI or SADs in our study were not reported in patient reports. However, the level of intubation experience and the nature of cardiac arrest cases such as minimising interruptions might contribute to SADs use in these cases.

Over the six years, we had only 24 patients from one hospital requiring initial advanced airway management in pre-hospital care and the other two hospitals had no patients. The small contribution of hospital-based EMS in initial response in KSA may explain the small sample size of our study, as the SRCA is responsible for initial response to all patients across the kingdom. However, Interfacility Transfers (IFT) are highly dependent on hospital-based EMS and the SRCA has no involvement in these transfers. This means that hospital-based paramedics should also focus on other required airway management skills for these transfers such as mechanical ventilation. In KSA, the most common adverse event during IFT was desaturation and most paramedics, when this event occurred, were seen to switch from mechanical ventilation to bag-valve mask ventilation (Alabdali et al., 2017). However, the reasons for these actions and their impact on outcomes are still unclear and require further assessment and investigation (Alabdali et al., 2017). Therefore, assessing other important airway management skills required for Saudi hospital-based paramedics during IFT is needed.

Our study is the first to assess the perception and success rate of pre-hospital advanced airway management in KSA. We had good response (70%) and completion (95%) rates for our questionnaire. However, we encountered some limitations that need to be highlighted. The retrospective design was a major limitation. We also had a small sample size in phase 1 which may impact the accuracy of our findings.

Our study had several implications. As we had a small sample size, further larger-scale prospective research including more hospitals and the SRCA to have a larger and more representative sample size is needed to assess the success rate of using different pre-hospital advanced airway management interventions in KSA. Future research looking into the impact of intubation experience on the success rate of pre-hospital intubation by paramedics is also needed. Furthermore, we did not assess the impact of such interventions on outcomes, which needs further assessment and investigation. Future studies assessing paramedics’ use of mechanical ventilation during IFT are needed to inform clinical practice about the existing challenges around paramedic-dependent IFT.

## Conclusion

This study is the first to assess the perception and success rate of paramedics’ use of advanced airway management in KSA. Most intubated cases were cardiac arrest cases. TI was used as the first choice for most patients requiring advanced airway management (16/24, 67%), and the remainder were managed using SADs (8/24, 33%). The paramedics’ use of TI had a success rate of 69% from the first two attempts compared to SADs (100%) from the first attempt. Most (80%) of the paramedics classified themselves as moderately or very competent in dealing with patients who required advanced airway management. However, most (80%) performed 1–5 TIs or SADs each year. The use of LMA was preferred by paramedics (63%) over other SADs. Further studies are needed to assess the success rate of different pre-hospital advanced airway management techniques on a larger scale, the impact of pre-hospital airway interventions on outcomes and the challenges of using mechanical ventilation by paramedics during IFT.

## Acknowledgements

We would like to thank all contributing centres, departments and paramedics working in all three hospitals for their cooperation and support.

## Author contributions

AmA, BA and NS designed the study protocol, collected, analysed and interpreted the data and drafted and edited the manuscript. AbA and MA helped to develop the study questionnaire and reviewed and edited the study protocol and manuscript drafts. NeA helped in ethical approval acquisition and reviewed the study protocol. NaA assisted in phase 1 data collection and coding and also in phase 2 through distributing the study questionnaire. SA supervised the study progress and manuscript development. All authors reviewed, edited and approved the final draft of the manuscript. AmA acts as the guarantor for this article.

## Conflict of interest

None declared.

## Data availability statement

The data that support the findings of this study are available from the corresponding author upon reasonable request.

## Ethics

Ethical approval from King Abdullah International Medical Research Center (KAIMRC) was obtained. In phase 2, written informed consent was obtained from participants.

## Funding

None.
